# Application of cold intolerance symptom severity questionnaire among vibration-exposed workers as a screening tool for the early detection of hand-arm vibration syndrome: a cross-sectional study

**DOI:** 10.1186/s40557-019-0284-x

**Published:** 2019-03-01

**Authors:** A Ram Kim, Dae Yun Kim, Ji Soo Kim, Heun Lee, Joo Hyun Sung, Cheolin Yoo

**Affiliations:** 1 Department of Occupational and Environmental Medicine, Ulsan University Hospital, University of Ulsan College of Medicine, 877 Bangeojinsunhwando-ro, Dong-gu, Ulsan, 44033 Republic of Korea; 2 Department of Occupational and Environmental Medicine, Gyeongsang National University College of Medicine, Gyeongsang National University Changwon Hospital, Institute of Health Science, 15, Jinju-daero 816beon-gil, Jinju-si, Gyeonsangnam-do 52727 Republic of Korea

**Keywords:** Hand-arm vibration syndrome, Cold intolerance, CISS questionnaire, Screening test

## Abstract

**Background:**

The detection rate of hand-arm vibration syndrome (HAVS) is very low in South Korea compared with other countries. The absence of uniform consensus and guidelines for diagnosing HAVS has been presumed to be one of the reasons. The HAVS has various manifestations including cold intolerance and its severity can be measured using the cold intolerance symptom severity (CISS) questionnaire. This study aimed to determine whether the CISS questionnaire, being used as a screening tool, can aid in the early detection of HAVS.

**Methods:**

A total of 76 male workers with vibration-induced symptoms were enrolled as the final study participants. To compare the CISS score of healthy individuals, 41 men who had never been exposed to local vibration were included in the study. In addition to the former medical questionnaire, the participants answered the CISS questionnaire. A statistical analysis was conducted to identify the association of CISS scores with vibration induced symptom and to determine its cut off value.

**Results:**

The reliability of the CISS questionnaire was proven to be good, with a total Cronbach’s alpha of 0.922. The mean CISS score of the exposed group increased in every vascular stage [stage 0 = 42.6 (18.5); stage 1 = 59.4 (14.1); and over stage 2 = 60.2 (21.6)]. They were significantly higher than that of the non-exposed group. The result was fairly consistent with those in the sensorineural stage. The sensitivity, specificity, positive predictive value (PPV), negative predictive value (NPV), and area under curve (AUC) of 30 were 88.5, 65.3, 76.1, 82.1 and 0.769, respectively. From the result of logistic regression, the adjusted odds ratio of both components increased by the CISS score grouped by 30s.

**Conclusions:**

The self-reported CISS questionnaire, used to measure the degree of cold intolerance, showed high agreement with the Stockholm classification of HAVS. Hence, we recommend the use of this questionnaire to assess the level of cold intolerance among vibration-exposed workers and detect individuals who are at risk of vibration-induced impairment with a cutoff value of 30.

**Trial registration:**

IRB No. 2018–07–040-001. Registered on 4 September 2018.

## Background

Hand-arm vibration syndrome (HAVS) is a potentially disabling condition comprising one or more specific neurological, vascular, and musculoskeletal features, associated with exposure to hand-held vibrating tools such as grinders [1]. The typical manifestation of HAVS is cold-induced vasospasm of the finger, which is often referred to as vibration-induced white finger [2]. Other symptoms include hypersensitivity to cold exposure and sensorineural symptoms such as tingling sensation, numbness, paresthesia, and sensory loss. These neurological features are known to precede vascular symptoms, which result in decrease in work ability and debilitation of daily life [3].

As there is no internationally agreed gold standard, the diagnosis of HAVS is based on the typical clinical features, history of exposure to vibration, and exclusion of other conditions [4]. Clinical assessment of vibration-induced impairment is classified based on the Stockholm workshop scale from 1986. According to the practical guidelines of the Korean employee health management 2015, the following initial tests should be conducted in vibration-exposed workers: work history investigation, peripheral circulation exam, neurological test, and muscular function test [5]. Based on the results, the doctor decides who among the workers needs further examination. This practical guideline published on 2015 reflects the actual condition of the health management system in South Korea, which only lists the exams without any organized or shared criteria that can be used to select workers requiring further evaluation.

In fact, according to the Ministry of Employment and Labor’s announcement in 2016, 32,217 workers were reported to be exposed to hand-transmitted vibration and only 14 of them were diagnosed with HAVS and compensated [6, 7]. From the meta-analysis performed on vibration-exposed workers in various countries, the incidence of vibration-induced white finger ranged from 13.8 to 55.7%, which was higher than that in South Korea [8]. The incidence of vibration-induced finger is dependent on the type of vibration tool and duration of exposure, because the degree of disability increases as the intensity and duration of vibration exposure increases [9–12]. According to ISO 5349:2001, when exposed to a vibration velocity of 10 m/s per day, 1 out of 10 workers whose working duration exceeds 6 years can eventually develop vibration-induced white finger [8, 13, 14]. One of the most common tools used in a typical industrial line in South Korea is grinders, and its velocity is about 5–10 m/s [15]. Based on this theory, we hypothesized that the use of inappropriate diagnostic systems or errors in occupational health exams conducted in vibration-exposed workers resulted in the underdiagnosis of vibration-induced disabilities, leading to the low incidence of HAVS in South Korea.

Cold intolerance is defined as an abnormal or exaggerated reaction to cold exposure of an injured part, causing discomfort or avoidance of cold [16]. As it is a common complaint following hand injuries including HAVS, cold intolerance had been frequently studied in conjunction with hand injuries and vibration-induced impairments [17–19]. An interesting fact is that cold intolerance showed a high agreement with Stockholm workshop classification [20]. Various questionnaires have been used to detect cold intolerance. Among those, the self-reported cold intolerance symptom severity (CISS) questionnaire proved to be a reliable, valid, and effective tool to check the degree of cold intolerance [21, 22]. It is important to assess vibration-exposed workers for cold intolerance since it may be an early neurological symptom of vibration-induced injury [18, 19]. Carlsson et al. reported that the cold intolerance identified by the CISS questionnaire was more apparent in patients with HAVS than in healthy individuals, including the severity and occurrence of symptoms and influence on daily life, disability, and health-related quality of life [18].

The current questionnaire administered to vibration-exposed workers as part of their occupational health exam includes various questions on the risk factors of HAVS. Unfortunately, it merely has ancillary role in helping doctors assess the vibration-exposed workers. As it cannot be quantified, these questionnaires could not be used as a screening tool nor act in consensus with the healthcare providers of vibration-exposed workers. We hypothesized that by applying the CISS questionnaire as part of the occupational health exam for vibration-exposed worker, we can quantify the degree of worker’s symptoms using the questionnaire and sort out people who need to take further objective exams to diagnose HAVS. Thus, it can make up for the weak point of the current system used in diagnosing HAVS. Hence, this study aimed to evaluate whether the CISS questionnaire is a valid screening tool that can be used for the early detection of HAVS and, if it is possible, to find out the appropriate cutoff value for vibration-exposed workers in South Korea.

## Methods

### Study participants

The study was performed by obtaining the history and health exam results of vibration-exposed workers who visited Ulsan University Hospital and underwent occupational health exam or consulted an occupational-environmental physician to evaluate the symptoms of HAVS from January 2011 to December 2016. The exposure group was defined as symptomatic workers who had been exposed to vibration by using vibration-inducing tools. The medical records of 91 individuals were reviewed. All participants were men and aged over 18 years. Seven participants who were not able to complete the questionnaire and eight who had a missing Stockholm stage were excluded. Finally, a total of 76 workers comprised the exposure group. On the contrary, the control group was composed of individuals who had never been exposed to hand-transmitted vibration. The CISS questionnaire scores of the exposed group and healthy individuals were compared. All participants in the control group were men and aged 20–60 years. Six of the total 40 compare group were excluded from the analysis due to history of hand trauma.

### Basic information survey and physical examination

Variables associated with vibration included age, present task, type of vibrating tool used, the duration of use, occupational posture, and safety equipment status [17]. Data regarding the participants’ medical history (hypertension, heart disease, diabetes mellitus, musculoskeletal disorders, trauma, etc.) and smoking history were collected because these variables may influence any current symptoms. After performing vascular and neurologic tests, the staging was conducted by a physician based on the Stockholm workshop scale.

In addition to the former questionnaire, the CISS questionnaire was applied. The first form of CISS questionnaire was cold sensitivity severity scale (CSS) invented by McCabe, which was updated 6 years later by Irwin into CISS questionnaire in Sweden. In 2006, Rujis et al. reported the modified version of CISS questionnaire [21, 23, 24]. The questionnaire is composed of six questions. The first question asks about the type of symptom an individual is experiencing and is not included in the scoring. The next five questions ask about the frequency of symptoms, time of occurrence, behavior change to ease the symptom, degree of symptom aggravation when performing certain activities, and how much the symptoms affected their daily life.

### Statistical analysis

First, an analysis was performed to check the reliability and validity of the CISS questionnaire. The reliability was assessed using Cronbach’s alpha scale. Content validity was assessed by the experts involved in the study, via literature review and comments from the participants of this study. Construct validity was investigated through factor analysis. Before performing the analysis, the exposed group was stratified using the Stockholm classification scale. Due to the limited number of samples with stage 3 and stage 4 vascular HAVS and stage 3 sensorineural HAVS, they were classified as over stage 2 group. In fact, each component was grouped into stage 0, 1, and over 2. We conducted a descriptive analysis to characterize samples of the study. The CISS score trend of the non-exposed group and the Stockholm stages of the exposed group were analyzed using ANOVA. To assess the association between CISS score and HAVS, logistic regression was conducted to calculate the univariate and multiple adjusted odds ratios (aORs) and 95% confidence intervals (CIs) while adjusting for potential confounding variables such as age, smoking status, duration of using vibration tools, and past medical history [17].

Several cutoff values for CISS questionnaire scores among normal population have been reported. Initially, the CISS score classification system was subdivided into mild (4–25), moderate (26–50), severe (51–75), and very severe (76–100) [21]. Recently, Sweden reported a cutoff value of 50, while Netherlands reported a cutoff value of 30 [18, 19, 21, 25]. Therefore, to find the appropriate cutoff value for screening HAVS in South Korean workers, we drew out three possible cutoff values: 20 and 30, which were estimated from the means and standard deviations of the exposure group, and 40 from the ROC curve.

Statistical analyses were performed using IBM SPSS Statistics for Windows version 21.0 (IBM SPSS Inc., Chicago, IL, USA), and *p*-values less than 0.05 were considered significant.

## Results

A total of 110 participants were enrolled in the study, of which 76 (69.1%) comprised the exposed group and 34 (30.9%) comprised the non-exposed group. Among the exposed group, 61 (80.3%) were diagnosed with HAVS. The mean (SD) age of the non-exposed group was 40.7 (10.3) years, which was significantly lower than that of the exposed group. A significant difference in the duration of vibration tool use and daily exposed hour in the sensorineural and vascular stages was observed in the exposed group. However, no trend was observed. Factors such as smoking status and past medical history had no significant difference in each stage (Table 1).

**Table 1:** General characteristic between non-exposed and exposed group based on Stockholm workshop stage

		Non-exposed (*n* = 34)	Exposed (*n* = 76)							
			Vascular stage			*p* value	Sensorineural stage			*p* value
			Stage 0 (*n* = 35)	Stage 1 (*n* = 19)	Stage 2 (*n* = 22)		Stage 0 (*n* = 25)	Stage 1 (*n* = 38)	Stage 2 (*n* = 13)	
Age		40.7 ± 10.3	47.5 ± 8.8	49.3 ± 9.3	52.2 ± 6.8	< 0.01	47.1 ± 9.5	49.7 ± 7.7	52.5 ± 8.2	< 0.01
Total exposure			225.6 ± 112.8	242.4 ± 106.0	244.2 ± 87.4	< 0.01	197.1 ± 103.1	252.7 ± 98.4	257.4 ± 105.3	< 0.01
Daily exposure			5.2 ± 2.9	6.7 ± 2.2	4.8 ± 3.2	< 0.01	5.7 ± 2.9	5.1 ± 3.0	6.2 ± 2.5	< 0.01
Non-smoker		22 (64.7)	24 (68.6)	13 (68.4)	11 (50.0)	0.505	20 (80.0)	22 (57.9)	6 (46.2)	0.160
Smoker		12 (35.3)	11 (31.4)	6 (31.6)	11 (50.0)		5 (20.0)	16 (42.1)	7 (53.8)	
Diabetes	No	30 (88.2)	35 (100)	19 (100)	22 (100)	0.037	25 (100)	38 (100)	13 (100)	0.032
	Yes	4 (11.8)	–	–	–		–	–	–	
Hypertension	No	31 (91.2)	31 (88.6)	16 (84.2)	20 (90.9)	0.864	23 (92.0)	32 (84.2)	12 (92.3)	0.771
	Yes	3 (8.8)	4 (11.4)	3 (15.8)	2 (9.1)		2 (8.0)	6 (15.8)	1 (7.7)	
Dyslipidemia	No	34 (100)	35 (100)	19 (100)	21 (95.5)	0.373	25 (100)	37 (97.4)	13 (100)	1.000
	Yes	–	–	–	1 (4.5)		–	1 (2.6)	–	

Unit: mean ± standard deviation, number (percentage)

*p*-value was calculated by ANOVA for continuous variables

*p*-value was calculated by chi-square test for categorical variables

The internal consistency of the questionnaire expressed by Cronbach’s alpha was very good. The total Cronbach’s alpha of all items in the translated questionnaires was 0.922. The construct validity of the questionnaire tested using factor analysis showed that the items in the CISS questionnaire were valid.

**Fig. 1:**
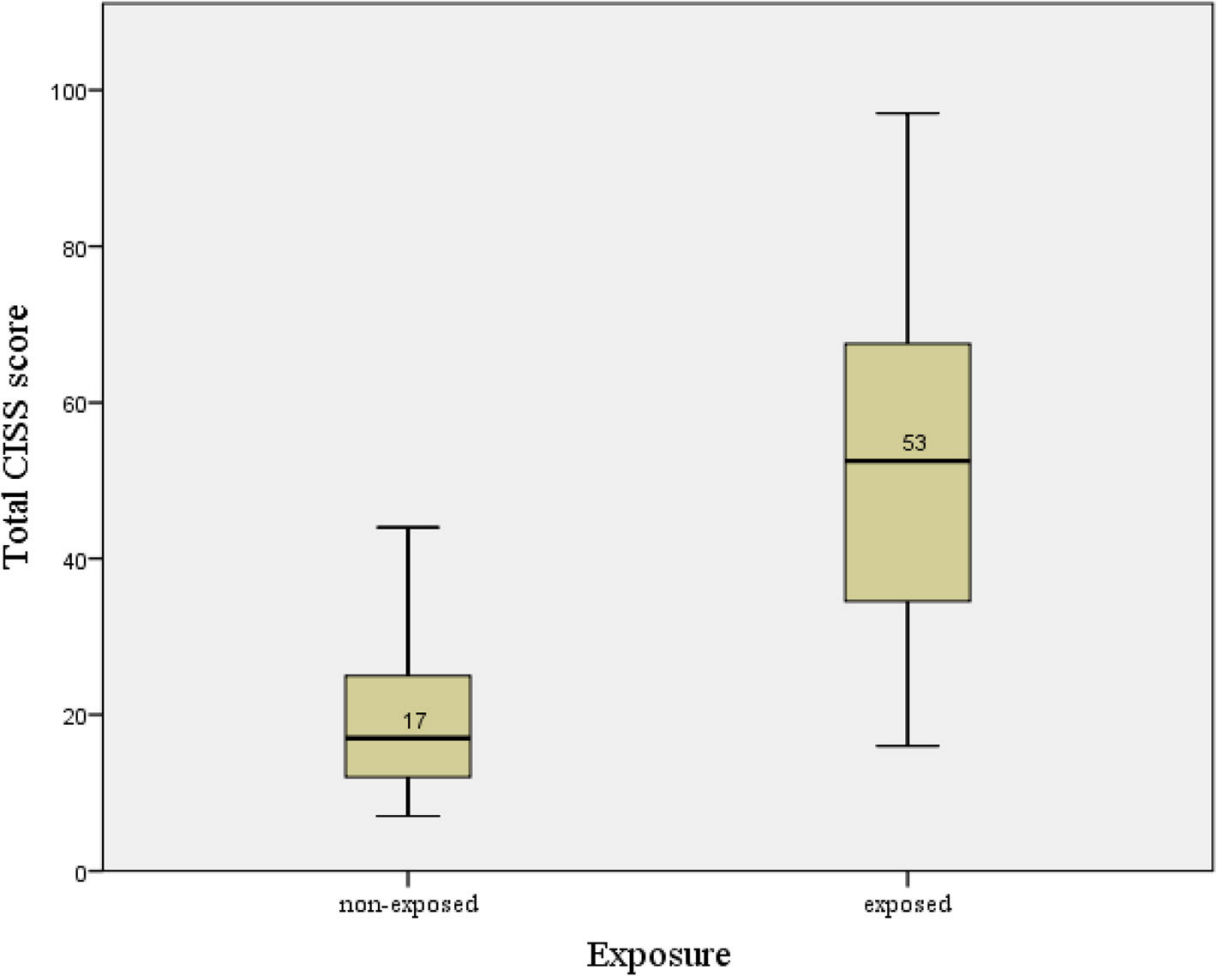
Comparison of total CISS score between vibration-exposed group versus non-exposed group. The line in the box represents the median value of total CISS score and the top and bottom of the box is the upper and lower quartile range

**Fig. 2:**
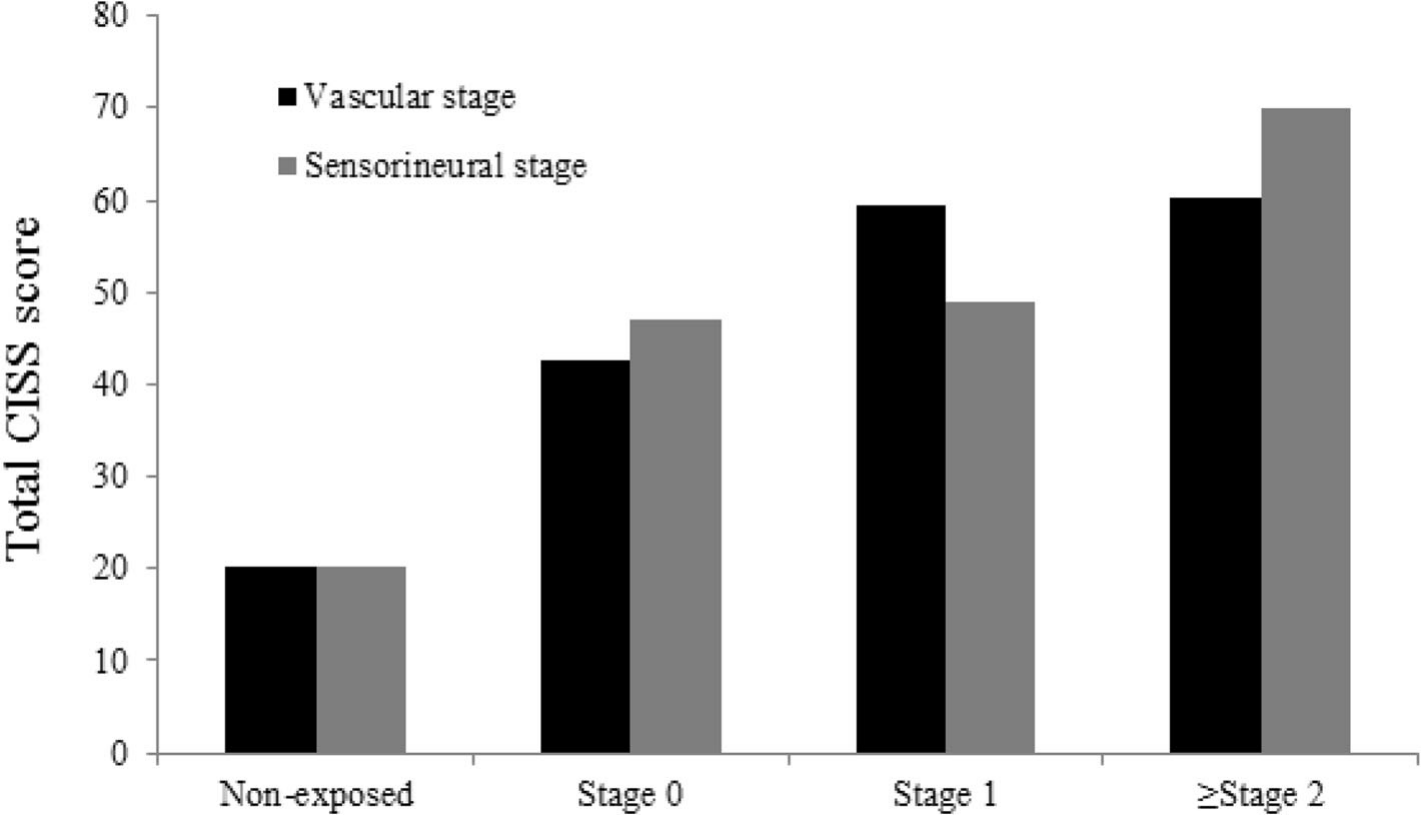
Pattern of total CISS score by both vascular and sensorineural component. This column graph shows mean total CISS score of non-exposed group and of the stages of vascular and sensorineural component. The mean total CISS score of both component is higher than that of the non-exposed group

The median total CISS score of the vibration exposed group was higher than that of the non-exposed group (Fig. 1). The CISS score pattern according to vascular and sensorineural stage is presented in Table 2 and expressed as means and standard deviations (SD). It was compared with that of the control group and their increasing pattern is depicted in a column graph (Fig. 2). In both components, the exposed group’s mean score was significantly higher than that of the non-exposed group, which was 20.3 (11.0). The mean (SD) CISS score of the exposed group increased in every vascular stage [stage 0 = 42.6 (18.5); stage 1 = 59.4 (14.1); and over stage 2 = 60.2 (21.6)]. They were significantly higher than those of the non-exposed group (mean = 20.6; SD = 11.0). The post-hoc result revealed the significant difference between the non-exposed group and stage 0 of the exposed group and between stages 0 and 1 and over stage 2. The result was fairly consistent with those in the sensorineural stage. The mean (SD) CISS score showed an increasing trend, 47.0 (18.3), 49.0 (19.4), and 70.1 (16.7). The post-hoc result, non-exposed group, and stage 1 of the sensorineural component were significantly different. However, unlike the vascular stage, no statistical significance was observed between stage 0 and stage 1.

**Table 2:** CISS score pattern according to the vascular and sensorineural stage

	Non-exposed^a^(*n* = 34)	Exposed (*n* = 76)			*p* value^‡^	Post-hoc comparison
		Stage 0^b^	Stage 1^c^	≥ Stage 2^d^		
Vascular stage	20.3 ± 11.0	42.6 ± 18.5 (*n* = 35)	59.4 ± 14.1 (*n* = 19)	60.2 ± 21.6 (*n* = 22)	< 0.01	a < b < c,d
Sensorineural stage		47.0 ± 18.3 (*n* = 25)	49.0 ± 19.4 (*n* = 38)	70.1 ± 16.7 (*n* = 13)	< 0.01	a < b,c < d

Unit: mean ± standard deviation, number (number of people included in each stage) ^‡^*p*-value was calculated by ANOVA

^ a,b,c,d^Each presented for the Post-hoc comparison which represents Non-exposed, Stage 0, Stage 1, ≥ Stage 2, respectively

**Fig. 3:**
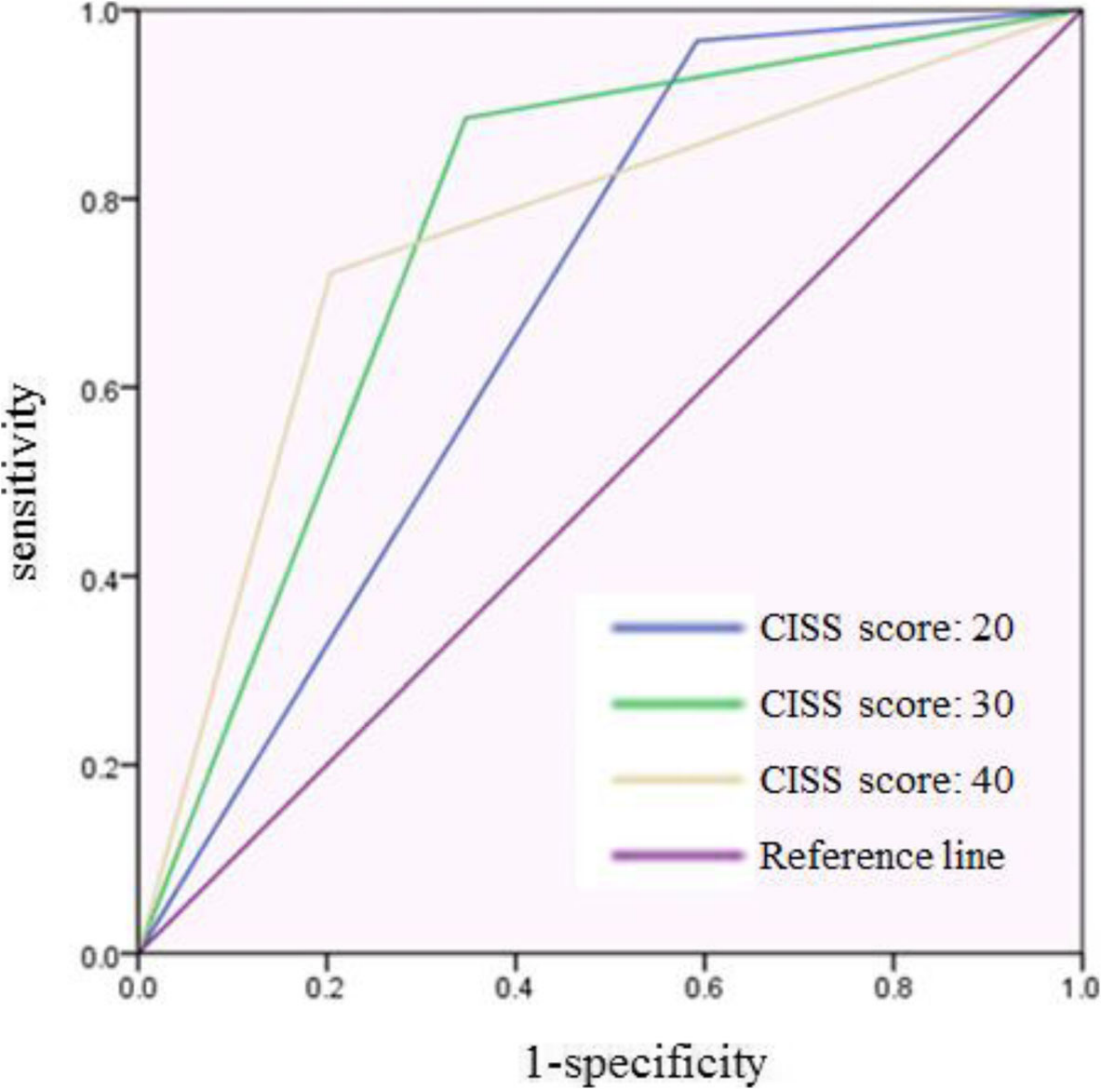
ROC curve of each estimated cutoff value. Blue line indicates the ROC curve of cutoff value 20. Green line indicates the ROC curve of cutoff value 30. Yellow line indicates the ROC curve of cutoff value 40. Purple line is the reference line

Table 3 shows the sensitivity, specificity, positive predictive value (PPV), and negative predictive value (NPV) of each estimated cutoff value. The ROC curve for each cutoff value is presented in Fig. 3. The area under the curve (AUC) values for each cutoff value were 0.688, 0.769, and 0.759 respectively, where 30 was considered as the best score but was not largely different from the score of 40.

**Table 3:** Test characteristics of proposed cutoff value of the CISS questionnaire to detect HAVS

Cut off value	Sensitivity	Specificity	PPV	NPV	AUC
20	96.7	40.8	81.5	90.1	0.688
30	88.5	65.3	76.1	82.1	0.769
40	72.1	79.6	67.0	69.6	0.759

Table 4 shows increasing pattern of OR and aOR by the CISS score in each vascular and sensorineural component of HAVS. We analyzed the vascular and sensorineural components separately to determine the relevance of CISS score with each component. The CISS score was categorized into 20s and 30s at each component of the Stockholm scale to compare which scoring group is more appropriate to assess HAVS. For the vascular component, when the baseline was set as the score group under 20, the OR and aOR of the score group from 21 to 40 were not considered significant. However, the OR and aOR of the 41–60 group [OR = 9.33 (95% CI = 1.88–46.35); aOR = 6.54 (95% CI = 1.15–37.24)] and over 61 group [OR = 46 (95% CI = 8.41–251.66); aOR = 24.34 (95% CI = 3.84–154.20)] significantly increased compared with the baseline, which indicates that the risk of HAVS increased as the CISS score increased. The result was more consistent when the baseline score was set to 30. The OR significantly increased as the CISS score increased [OR of the 31–60 group = 13.54 (95% CI = 2.90–63.28); OR of the over 61 group = 82.42 (95% CI = 15.38–441.54)] and remained significant even after adjusting the possible confounding variables.

**Table 4:** Odds ratio and adjusted odds ratio of CISS score between non-exposed and exposed group

		CISS score	*N*	Before adjustment		After adjustment	
				OR^a^	95% CI	aOR^b^	95% CI
Vascular	CISS score grouped by 20	Under 20	23	1.00		1.00	
		21–40	33	1.29	0.20**–**8.35	0.96	0.13**–**6.90
		41–60	27	9.33	1.88**–**46.35	6.54	1.15**–**37.24
		Over 61	27	46.00	8.41**–**251.66	24.34	3.84**–**154.20
	CISS score grouped by 30	Under 30	41	1.00		1.00	
		31–60	42	13.54	2.90**–**63.28	10.47	2.08**–**52.70
		Over 61	27	82.42	15.38**–**441.54	47.38	8.07**–**278.19
Sensorineural	CISS score grouped by 20	Under 20	23	1.00		1.00	
		21–40	33	2.25	0.60**–**8.40	2.00	0.33**–**12.16
		41–60	27	7.07	1.98**–**25.28	3.62	0.63**–**20.68
		Over 61	26	14.44	3.78**–**55.19	4.47	0.76**–**26.36
	CISS score grouped by 30	Under 30	41	1.00		1.00	
		31–60	42	6.51	2.39**–**17.73	4.96	1.32**–**18.71
		Over 61	27	14.25	4.53**–**44.82	5.29	1.27**–**22.07

^a^Odds ratio was calculated by logistic regression analysis

^b^Adjusted odds ratio was calculated by multiple logistic regression analysis after adjusting age, total period of using vibration-inducing tools, current smoking status, and past medical history

Besides, the sensorineural component gained a different result. When the score was categorized as 20s, there was no significant increase in the OR or aOR in any score group. However, when we assigned the under 30 group as baseline, the OR of the 31–60 group and over 61 group showed an increasing pattern [OR of 31–60 score group = 6.51 (95% CI = 2.39–17.73); OR of over 61 group = 14.25 (95% CI = 4.53–44.82)] and remained significant even after adjustment of confounding variables.

## Discussion

The present study shows an association between CISS score and HAVS. The CISS score reflects the degree of cold intolerance, while the Stockholm stage reflects the severity of the disease. From the increasing pattern of the mean CISS score based on the Stockholm stage (Table 1), it can be inferred that cold intolerance is related to disease severity in both vascular and sensorineural components of HAVS. This also corresponds to the complex pathophysiology of cold intolerance. Cold intolerance, which is the mainstay of CISS questionnaire, is usually seen among the hand-injured patients. This debilitating symptom persists, causing a serious problem as it affects an individual’s daily life and performance at work [16, 19, 21, 26, 27]. The mechanism of cold sensitivity is still under debate, to identify whether it is caused by a thermoregulatory dysfunction or by a neurologic condition that triggers neuropathic pain, which is again applicable to that of HAVS. Similar to the complex mechanism of cold intolerance, patients with HAVS may present with various organ dysfunctions. Moreover, its mechanism is known to be complex, with impairment of the sympathetic nervous system and dysfunction of the vascular system [16, 28–30]. Obviously, cold intolerance is an important manifestation of HAVS as in other hand injuries, which many patients experience [31]. Additionally, results showed that the association is more distinct in the vascular component than in the sensorineural component. This is because, generally, peripheral neuropathic symptoms due to local vibration exposure are known to precede peripheral vascular symptoms. Thus, patients with higher vascular stage may have the peripheral neuropathic disorder already and thus experience more severe cold intolerance [3].

As there is no gold standard diagnostic tool for HAVS, the physical exam by the experienced occupational doctor and history of occupational exposure has an important role in diagnosing HAVS [4]. Testing for neurology and motor as well as vascular function is ancillary to that. The validity of testing tools had different values at each study, and there are no specific standards to date [4, 31–35]. The cold provocation test is known to be the objective test to assess for presence of vascular impairment in patients with HAVS, but there is variability among the studies [34, 36, 37]. About the neurologic impairment, a previous study reported about the objective tests but no consensus has been established yet [31, 36, 38]. Thus, the diagnosis of HAVS is largely dependent on the patients’ subjective complaint and physical exam performed by an experienced occupational specialist. The questionnaires included in the recent occupational exams for vibration-induced workers contain various questions that can help in diagnosing HAVS. However, each item cannot be quantified, which makes it difficult to use as a screening questionnaire.

In this background, the need for developing consensus among the occupational specialist has emerged. We suggest using CISS questionnaire as a screening tool for HAVS. The CISS questionnaire can be used to quantify the subjective symptoms by computing the total score. Health care managers in the vibration-exposed industry require all workers to fill out the questionnaires periodically to identify those who need further evaluation, which enhances accessibility to diagnosis of vibration-induced impairments. In addition, occupational health management organizations can use the questionnaire as an evidence to conduct expensive tests to confirm HAVS and provide additional objective data in physical exams. Moreover, the specific symptom of HAVS, also known as vibration-induced white finger, is rarely reported in warm climate but neurologic symptoms such as tingling sensation and numbness have been reported even in warm climate [39, 40]. While vibration induced white finger is rarely observed during summer in South Korea, cold intolerance can be triggered by factors such as humidity, rain, and windy weather [18]. In this sense, the CISS questionnaire can be applied regardless of the season, making it more useful.

To our knowledge, this is the first study conducted in search for the cutoff value that can be used in a questionnaire to screen for HAVS and test for its validity and reliability. By comparing the sensitivity, specificity, PPVs, and NPVs among the three estimated cutoff values, scores above 30 or 40 would indicate the need for further evaluation. Supporting this suggestion, the risk of HAVS is well expressed when the CISS score is grouped by 30s than 20s, which infers that a CISS score of 30 would be more appropriate to predict the people possibly having HAVS. Additionally, as it is said above, we strongly doubt that the incidence of HAVS has been underestimated in South Korea considering the prevalence of HAVS in other country. In this sense, we suggest 30 as the proper cutoff value to avoid missing the detection of vibration-induced disorders among the workers for now.

This study has several limitations. First, this study is a cross-sectional study, which makes it hard to determine the causal relationship between cold intolerance proven by the CISS questionnaire and HAVS. Second, there could have been a recall bias since CISS questionnaire is a self-reported questionnaire. Moreover, there is a limitation within the questionnaire itself. The CISS questionnaire used in this study is not the one recently modified. The recent version of the CISS questionnaire has additional response options in questions number two and three, allowing the minimum total score of zero and making the symptoms of the unexposed group to be more clearly identified. Additionally, the contents of the CISS questionnaire are mostly about the effects of cold intolerance on daily life. To use it as a screening tool to evaluate the effects of cold intolerance on performance at work, it is advisable to add the items that focus more on identifying the discomforts experienced by the vibration exposed group than focusing on the daily discomfort. Definitely, future studies must be conducted using the revised CISS questionnaire to confirm its role as a screening tool. Finally, there are no definite objective tools to compare the sensitivity and specificity of the questionnaire in diagnosing HAVS. There have been a number of studies in search of confirmative tests to diagnose HAVS. However, the gold standard of diagnosis remains unknown. In the future, we hope to conduct a study that evaluates the correlation between the CISS scores and other objective diagnosing tools used to identify the degree of vascular or sensorineural impairment in vibration exposed workers.

Nevertheless, the significance of this study is that this is the first study to test the validity and reliability of the CISS questionnaire and to adopt the CISS questionnaire as a tool to screen individuals with HAVS in South Korea. As mentioned above, cold intolerance is one of the most debilitating complications of hand injuries experienced by several patients, including HAVS. Cold intolerance is a symptom that cannot be completely eliminated. Many studies have reported the practice of lifestyle modification to improve symptoms and the study is still ongoing. Only early diagnosis can make early intervention possible, and only early intervention (e.g., stop vibration exposure) can stop the progression of the symptom.

Future studies are needed to modify the questionnaire according to the purpose of using it as a screening tool for the early detection of patients with HAVS. Many studies using the CISS questionnaire were conducted in conjunction with the potential work exposure scale (PWES) [22]. The PWES includes questions about the exposure of hands to cold in the workplace. If it is used along with the CISS questionnaire in the workplace, it will be more helpful in caring for vibration-exposed workers. Finally, other than using the CISS questionnaire as a screening tool, it can also be used to detect people who have cold intolerance as the symptom persists for a long time, causing discomfort in their daily life as well as in their working life and educate them how to manage the symptom and ways to relieve the symptom.

## Conclusion

The prevalence of HAVS in South Korea is low compared to other countries. As there is no gold standard, doctor’s subjective examination is important in diagnosing HAVS. The detection rate of HAVS may differ according to the degree of attention paid by physicians due to the absence of the definitive diagnostic tool. This may be one of the reasons for the undervaluation of HAVS. The self-reported CISS questionnaire measuring the degree of the cold intolerance shows high agreement with the Stockholm classification scale of HAVS. Hence, we recommend the use of the CISS questionnaire as a screening tool among the vibration-exposed workers and for the early diagnosis of people at risk of vibration-induced impairment, with a cutoff value of 30.

## Abbreviations

aOR: Adjusted odds ratio
AUC: Area under the curve
CI: Confidence intervals
CISS: Cold intolerance symptom severity
HAVS: Hand-arm vibration syndrome
NPV: Negative predictive value
OR: Odds ratio
PPV: Positive predictive value
PWES: Potential work exposure scale
SD: Standard deviation

## References

[1] Lawson IJ, McGeoch KL (2003). A medical assessment process for a large volume of medico-legal compensation claims for hand-arm vibration syndrome. Occup Med (Lond).

[2] Heaver C, Goonetilleke KS, Ferguson H, Shiralkar S (2011). Hand-arm vibration syndrome: a common occupational hazard in industrialized countries. J Hand Surg Eur Vol.

[3] Nilsson T, Wahlstrom J, Burstrom L (2017). Hand-arm vibration and the risk of vascular and neurological diseases-a systematic review and meta-analysis. PLoS One.

[4] Mahbub M, Harada N (2011). Review of different quantification methods for the diagnosis of digital vascular abnormalities in hand-arm vibration syndrome. J Occup Health.

[5] Korea Occupational Safety and Health Agency (KOSHA) (2015). Health examination guideline for Korean worker.

[6] Ministry of Employment and Labor: Workers’ Health Examination Result in South Korea, 2016. https://www.moel.go.kr/info/publicdata/majorpublish/majorPublishList.do. Accessed 29 June 2018.

[7] Ministry of Employment and Labor: Analysis of Current Occupational Injury in South Korea, 2016. https://www.moel.go.kr/info/publicdata/majorpublish/majorPublishList.do. Accessed 29 June 2018.

[8] Bovenzi M (2012). Epidemiological evidence for new frequency weightings of hand-transmitted vibration. Ind Health.

[9] Brammer AJ. Dose-response relationships for hand-transmitted vibration. Scand J Work Environ Health. 1986:284–8 PMID: 3775313. 10.5271/sjweh.2139 3775313

[10] Bovenzi M (1998). Exposure-response relationship in the hand-arm vibration syndrome: an overview of current epidemiology research. Int Arch Occup Environ Health.

[11] Bovenzi M (2010). A prospective cohort study of exposure-response relationship for vibration-induced white finger. Occup Environ Med.

[12] Edlund M, Burstrom L, Gerhardsson L, Lundstrom R, Nilsson T, Sanden H (2014). A prospective cohort study investigating an exposure-response relationship among vibration-exposed male workers with numbness of the hands. Scand J Work Environ Health.

[13] ISO (2001). Mechanical vibration-measurement and evaluation of human exposure to hand-transmitted vibration.

[14] ISO (1986). Mechanical vibration-Guidelines for the measurement and the assessment of human exposure to hand-transmitted vibration.

[15] Yoo C, Lee JH, Lee CR, Kim Y, Lee H, Choi Y (2005). Occupational hand-arm vibration syndrome in Korea. Int Arch Occup Environ Health.

[16] Smits E. Cold intolerance: from thermoregulation to nerve innervation 2014.

[17] Craigen M, Kleinert JM, Crain GM, McCabe SJ (1999). Patient and injury characteristics in the development of cold sensitivity of the hand: a prospective cohort study. J Hand Surg Am.

[18] Carlsson IK, Rosen B, Dahlin LB (2010). Self-reported cold sensitivity in normal subjects and in patients with traumatic hand injuries or hand-arm vibration syndrome. BMC Musculoskelet Disord.

[19] Carlsson IK, Dahlin LB (2014). Self-reported cold sensitivity in patients with traumatic hand injuries or hand-arm vibration syndrome - an eight year follow up. BMC Musculoskelet Disord.

[20] Cederlund R, Iwarsson S, Lundborg G (2003). Hand function tests and questions on hand symptoms as related to the Stockholm workshop scales for diagnosis of hand-arm vibration syndrome. J Hand Surg Br.

[21] Ruijs AC, Jaquet JB, Daanen HA, Hovius SE (2006). Cold intolerance of the hand measured by the CISS questionnaire in a normative study population. J Hand Surg Br..

[22] Carlsson I, Cederlund R, Hoglund P, Lundborg G, Rosen B (2008). Hand injuries and cold sensitivity: reliability and validity of cold sensitivity questionnaires. Disabil Rehabil.

[23] McCabe SJ, Mizgala C, Glickman L (1991). The measurement of cold sensitivity of the hand. J Hand Surg Am..

[24] Irwin MS, Gilbert SE, Terenghi G, Smith RW, Green CJ (1997). Cold intolerance following peripheral nerve injury. Natural history and factors predicting severity of symptoms. J Hand Surg Br..

[25] Carlsson IK, Nilsson JA, Dahlin LB (2010). Cut-off value for self-reported abnormal cold sensitivity and predictors for abnormality and severity in hand injuries. J Hand Surg Eur Vol.

[26] Traynor R, MacDermid JC (2008). Immersion in cold-water evaluation (ICE) and self-reported cold intolerance are reliable but unrelated measures. Hand (N Y).

[27] Wendt M, Novak CB, Anastakis DJ (2018). Prevalence of cold sensitivity in upper extremity nerve compression syndromes. J Hand Surg Eur Vol.

[28] Isogai N, Fukunishi K, Kamiishi H (1995). Patterns of thermoregulation associated with cold intolerance after digital replantation. Microsurgery.

[29] Stoyneva Z, Lyapina M, Tzvetkov D, Vodenicharov E (2003). Current pathophysiological views on vibration-induced Raynaud’s phenomenon. Cardiovasc Res.

[30] Ruijs AC, Jaquet JB, van Riel WG, Daanen HA, Hovius SE (2007). Cold intolerance following median and ulnar nerve injuries: prognosis and predictors. J Hand Surg Eur Vol.

[31] Lander L, Lou W, House R (2007). Nerve conduction studies and current perception thresholds in workers assessed for hand-arm vibration syndrome. Occup Med (Lond).

[32] Pelmear PL, Kusiak R (1994). Clinical assessment of hand-arm vibration syndrome. Nagoya J Med Sci.

[33] Coughlin PA, Chetter IC, Kent PJ, Kester RC (2001). The analysis of sensitivity, specificity, positive predictive value and negative predictive value of cold provocation thermography in the objective diagnosis of the hand-arm vibration syndrome. Occup Med (Lond).

[34] Poole K, Elms J, Mason HJ (2004). The diagnostic value of finger systolic blood pressure and cold-provocation testing for the vascular component of hand-arm vibration syndrome in health surveillance. Occup Med (Lond).

[35] Rolke R, Rolke S, Vogt T, Birklein F, Geber C, Treede RD (2013). Hand-arm vibration syndrome: clinical characteristics, conventional electrophysiology and quantitative sensory testing. Clin Neurophysiol.

[36] Kook YJ, Heun L, Ri CN, Hwan KS, Wook PH, Ho LJ (2009). Early objectified detection method of sensorineural component in hand arm vibration syndrome. J Occup Environ Med.

[37] Ye Y, Griffin MJ (2016). Assessment of two alternative standardised tests for the vascular component of the hand-arm vibration syndrome (HAVS). Occup Environ Med.

[38] Falkiner S (2003). Diagnosis and treatment of hand-arm vibration syndrome and its relationship to carpal tunnel syndrome. Aust Fam Physician.

[39] Su AT, Maeda S, Fukumoto J, Darus A, Hoe VC, Miyai N (2013). Dose-response relationship between hand-transmitted vibration and hand-arm vibration syndrome in a tropical environment. Occup Environ Med.

[40] Su AT, Darus A, Bulgiba A, Maeda S, Miyashita K (2012). The clinical features of hand-arm vibration syndrome in a warm environment--a review of the literature. J Occup Health.

